# Discovery of novel treponemes associated with pododermatitis in elk (*Cervus canadensis*)

**DOI:** 10.1128/aem.00105-24

**Published:** 2024-05-14

**Authors:** Sushanta Deb, Margaret A. Wild, Thomas LeClair, Devendra H. Shah

**Affiliations:** 1 Department of Veterinary Microbiology and Pathology, College of Veterinary Medicine, Washington State University, Pullman, Washington, USA; 2 School of Veterinary Medicine, Texas Tech University, Amarillo, Texas, USA; INRS Armand-Frappier Sante Biotechnologie Research Centre, Laval, Quebec, Canada

**Keywords:** *Treponema*, elk, hoof disease, metagenomics, pododermatitis, comparative genomics

## Abstract

Pododermatitis, also known as treponeme-associated hoof disease (TAHD), presents a significant challenge to elk (*Cervus canadensis*) populations in the northwestern USA, with *Treponema* spp. consistently implicated in the lesion development. However, identifying species-specific *Treponema* strains from these lesions is hindered by its culture recalcitrance and limited genomic information. This study utilized shotgun sequencing, *in silico* genome reconstruction, and comparative genomics as a culture-independent approach to identify metagenome-assembled *Treponema* genomes (MATGs) from skin scraping samples collected from captive elk experimentally challenged with TAHD. The genomic analysis revealed 10 new MATGs, with 6 representing novel genomospecies associated with pododermatitis in elk and 4 corresponding to previously identified species*—Treponema pedis* and *Treponema phagedenis*. Importantly, genomic signatures of novel genomospecies identified in this study were consistently detected in biopsy samples of free-ranging elk diagnosed with TAHD, indicating a potential etiologic association. Comparative metabolic profiling of the MATGs against other *Treponema* genomes showed a distinct metabolic profile, suggesting potential host adaptation or geographic uniqueness of these newly identified genomospecies. The discovery of novel *Treponema* genomospecies enhances our understanding of the pathogenesis of pododermatitis and lays the foundation for the development of improved molecular surveillance tools to monitor and manage the disease in free-ranging elk.

**IMPORTANCE:**

*Treponema* spp. play an important role in the development of pododermatitis in free-ranging elk; however, the species-specific detection of *Treponema* from pododermatitis lesions is challenging due to culture recalcitrance and limited genomic information. The study utilized shotgun sequencing and *in silico* genome reconstruction to identify novel *Treponema* genomospecies from elk with pododermatitis. The discovery of the novel *Treponema* species opens new avenues to develop molecular diagnostic and epidemiologic tools for the surveillance of pododermatitis in elk. These findings significantly enhance our understanding of the genomic landscape of the *Treponemataceae* consortium while offering valuable insights into the etiology and pathogenesis of emerging pododermatitis in elk populations.

## INTRODUCTION

An emerging hoof disease of free-ranging elk (*Cervus canadensis*) in the northwestern USA is characterized by severe pododermatitis associated with lameness and debilitation (1, 2). Gross lesions typically originate as cutaneous ulcerations in the interdigital space (IDS), and progress to severe ulceration and necrosis of the IDS, frequently extending to the heel bulb and resulting in sloughing of the hoof capsule (1, 3). Histologically, pododermatitis in elk exhibits suppurative inflammation with the consistent presence of invasive spiral-shaped bacteria typical of spirochetes (1). Characteristic histologic changes within the elk pododermatitis along with the presence of *Treponema* spp. exhibit similarities to bovine digital dermatitis (BDD) and contagious ovine digital dermatitis (CODD) in domestic livestock (1, 4)⁠. The consistent identification of lesion-associated *Treponema* spp., a member of the family *Treponemataceae* (formerly *Spirochaetaceae*), led to the designation of treponeme-associated hoof disease (TAHD) of elk (1, 4, 5). Although the presence of *Treponema* spp. is characteristic of BDD, CODD, and TAHD, and is likely the primary agent associated with these diseases, there is speculation that each of these diseases may involve multiple bacterial species (4, 6, 7).

In one study, *Treponema* spp. representing the three classical BDD phylotypes [*Treponema medium*/*Treponema vincentii*-like, *Treponema phagedenis*-like, and *Treponema denticola/Treponema putidum*-like (8)] were identified by PCR on isolates cultured using specialized techniques from a small number of samples from elk hooves (4). However, the isolation of fastidious *Treponema* spp. in pure culture is challenging because hoof samples are frequently tainted with soil, feces, and various other environmental microorganisms. Moreover, the investigation of diseases in free-ranging wildlife such as elk is further complicated by difficulties in obtaining fresh tissues that maintain the viability of *Treponema* spp. from a sizeable sample group. Therefore, most epidemiologic investigations of hoof disease in elk have relied on culture-independent techniques, such as 16S rRNA amplicon sequencing, to detect *Treponema* spp. (2, 3, 9)⁠. In our recent report, we discovered that in addition to the well-known treponemal phylotypes, partial sequencing of the 16S rRNA amplicons identified distinct signatures of previously uncultured *Treponemataceae,* including *Treponema* sp. clone 19 (PT19), from hooves of free-ranging elk diagnosed with TAHD (2). These novel signatures of uncultured spirochetes were also detected in early lesions, and before the development of graded TAHD lesions, in experimentally challenged captive elk (3)⁠. Signatures of uncultured PT19 were previously detected from BDD lesions in Norwegian cattle (10)⁠. In the last decade, other novel uncultured *Treponema* spp. have also been detected within BDD lesions from domestic cattle (6, 11, 12). Collectively, these findings have led to the hypothesis that PT19, and perhaps other novel spirochetes, are likely associated with TAHD in free-ranging elk (3). A few complete genome sequences of *Treponema* spp. cultured from BDD lesions from cattle are now publicly available (13, 14); however, the genome sequences of *Treponema* spp. detected within TAHD lesions in elk are not yet available. Therefore, the identity of these uncultured spirochetes remains obscure. This lack of genomic information poses significant limitations to etiological and epidemiological investigations of pododermatitis in elk.

Genome reconstruction approaches hold promise for investigating wildlife diseases where the etiology of disease is poorly understood, and sample quality is often compromised for routine microbiological processing especially when infectious agents, such as *Treponema*, are difficult to culture. Construction and comprehensive characterization of genomes of microbiologically fastidious *Treponema* spp. detected in TAHD lesions (2, 3) is fundamental to epidemiologic investigations, the development of improved tests for reliable diagnosis, and routine monitoring and surveillance of TAHD. Shotgun sequencing coupled with *in silico* genome reconstruction offers a viable alternative for novel pathogen discovery to improve our understanding of the etiology and pathogenesis of pododermatitis in elk.

The objective of this study was to construct and characterize the metagenome-assembled genomes (MAGs) of fastidious and culture-recalcitrant *Treponema* spp. from elk with TAHD lesions that were previously suspected to carry novel *Treponema* spp. signatures based on 16S rRNA amplicon sequencing (2, 3)⁠. To accomplish this, we developed a complete bioinformatics analysis pipeline to construct MAGs of culture-recalcitrant *Treponema* from shotgun metagenome data obtained from hooves of elk with TAHD. We then performed a comprehensive comparative genomics analysis of MAGs to identify novel genomospecies of *Treponemataceae* involved in pododermatitis in elk. We also demonstrate that multiple *Treponema* spp., including the novel genomospecies identified in this study, are indeed associated with pododermatitis in free-ranging elk.

## MATERIALS AND METHODS

### Elk hoof samples for shotgun sequencing

Nine elk hoof samples including seven biopsies from free-ranging elk and two skin scrapings from experimentally challenged captive elk collected and archived from previous studies were utilized in this study (2, 3). Biopsies were collected, post-mortem, from elk that were harvested or culled for management purposes by state wildlife agencies and, thus, were exempt from Institutional Animal Care and Use Committee review. These samples were selected for shotgun sequencing because 16S rRNA sequencing revealed that each had tested positive for the presence of unclassified *Treponemataceae* signatures, particularly PT19 (2, 3), raising the possibility that unique *Treponema* spp. associated with early lesions were likely present. The biopsy samples originated from the IDS of hooves of free-ranging elk previously submitted to the Washington Animal Disease Diagnostic Laboratory (Pullman, WA) for TAHD surveillance. Samples were from elk with abnormal hooves in areas where TAHD occurs in Washington, Idaho, and California (2)⁠. Multiple feet from each elk were examined histologically and TAHD was confirmed in every case; however, biopsies did not originate from a TAHD-confirmed foot in every case (Table S1). Biopsies were placed in Allprotect Tissue Reagent (Qiagen, Germantown, Maryland, USA) and frozen at –80 C until processed for genomic DNA (gDNA) extraction as previously described (2). The two skin scrapings selected for the current study originated from two captive experimental research elk that were sampled 98 days following the initial challenge with inoculum prepared from the hooves of TAHD-affected free-ranging elk from Washington as previously reported (3)⁠. Scrapings were collected from the IDS of feet with mild gross changes that did not meet the criteria for a graded lesion; however, each experimental elk exhibited an apparent grade I lesion on another foot at the time of sampling. The lesions on the experimental elk progressed and TAHD was confirmed by histologic examination of lesion biopsies (3) (Table S1). Using 16S rRNA amplicon sequencing, the unclassified *Treponemataceae* signatures, particularly PT19, were detected in both skin scrapings, which corroborated with the detection of these signatures in biopsies from free-ranging elk (2). However, the unclassified *Treponemataceae* signatures were not detected in these elk before the experimental challenge or in the control elk used in that study (3). Skin scrapings from experimental elk were placed in sterile vials and frozen at −80°C until processed for gDNA extraction as previously described (3).

### Shotgun metagenome sequencing of elk hoof DNA

An aliquot of gDNA was submitted to Novogene (Novogene Corporation Inc., Sacramento, California, USA) for shotgun sequencing using the Illumina platform with the objective of obtaining microbial reads for reconstructing potentially novel MAGs of *Treponema* spp. (hereafter referred to as MATGs) likely associated with early TAHD lesions. The sequencing libraries were constructed by random shearing of gDNA using NEBNext Ultra DNA Library Prep Kit (Illumina, USA), resulting in the generation of fragments with an approximate length of 350 base pairs (bp) with dA-tailing and NEBNext adapter ligation. Base calling was performed using the Illumina real-time analysis (RTA), thereby generating paired-end sequence reads with 150 bp length at each end. The resulting reads were trimmed, filtered, and deduplicated using the fastp tool with the following parameters “-l 100 --dedup --dup_calc_accuracy 6” (15). Reads with Phred score <25 and a length <150 bp were discarded from downstream processing. Host reads were removed by mapping to the elk reference genome (NCBI assembly accession GCF_019320065.1) using a Bowtie 2 aligner (16, 17). Host-subtracted reads were assembled into metagenomes using the Megahit program (v.1.2.9) (18). Following assembly, reads were individually mapped using Burrows-Wheeler Aligner to the metagenomic contigs for each sample (19), and mapping files were generated with SAM tools (20)⁠. Contigs ≥1,500 bp were selected for downstream processing using four different binning algorithms, including BinSanity v.0.2.6.1 (21), CONCOCT (22)⁠, MaxBin 2.0 v.2.2.4 (23), and MetaBAT 2 v.2.12.1 (24). To ensure that genome bins acquired from the binning method contained non-redundant MATGs, they were individually dereplicated using the DAS_Tool to obtain quality-filtered consensus MATGs (25)⁠. The bin quality and completeness of MATGs were evaluated based on the number of single-copy marker genes using CheckM v.1.0.7 program (26). MATGs with ≥90% completeness and ≤3% contamination were selected to forecast the taxonomic assignment using the taxonomic classification procedure of GTDB-Tk v.0.2.1 (27). MATGs of *Treponema* species curated from elk samples were annotated using the NCBI PGAP pipeline (28)⁠. To estimate the relative abundance of spirochetes, shotgun read sequences for each sample were mapped against the standard Kraken database. NCBI accession numbers were assigned to each mapped sequence followed by the assignment of taxonomy using Kraken 2 and KrakenTools software suite (29–31) with a confidence score of 0.5. To determine the relative abundance of novel MATG-specific reads in each sample, we performed back mapping of shotgun sequencing reads against each MATG using the script map-bowtie2-markduplicates.sh (32).

### Core gene phylogeny of MATGs from elk hooves

To identify core gene content, draft and complete genome sequences of 74 non-*Treponema pallidum Treponema* species along with one *T. pallidum* Str. SamoaD (accession number CP002374.1), as an outgroup, were retrieved from NCBI GenBank database (accessed 18 March 2023) (Table S2). Contamination and completeness of genomes retrieved from the NCBI GenBank database were estimated using CheckM v.1.1.3 (26)⁠. Genomes with completeness >90% and contamination <3% were included for the downstream analysis (33). Single-copy core genes with an *e*-value of 1.0 e − 15 were identified using best-bidirectional hits through genomic comparison (34, 35). The core gene hits were selected with ≥95% sequence identity and ≥85% alignment coverage. Non-recombinant core gene families were included for the construction of a maximum likelihood phylogenetic tree using RAxML v.8 (36) under Gamma + General Time Reversible model (37). To identify recombinant genes, the PhiTest feature included in the PhiPack program was employed (38)⁠.

### Genomic relatedness, functional profiling, and genomic clustering of MATGs from elk hooves

The taxonomic boundaries and genomic similarities of MATGs constructed from elk hooves were determined by calculating and comparing the average nucleotide identity (ANI) values using the Python package pyani.py (39) and average amino acid identity (AAI) using the “aai_wf” function implemented in CompareM program (v.0.1.2) with the default parameters (40). Conserved proteins were identified using a BLASTP match (*e*-value <1e − 5, sequence identity of >40%, and alignable area of the query sequence >50%) (41). The percentage of conserved proteins (POCP) between two genomes was calculated using the formula [(C1 + C2) / (T1 + T2)] * 100%, where C1 and C2 represent the number of conserved proteins in the two genomes under comparison and T1 and T2 represent the total number of proteins in two genomes being compared (41). A POCP value of ≥50% was considered as a criterion to taxonomically classify genomes within the same genus. ANI and AAI values ≥95% were used to taxonomically classify genomes within the same species (42, 43). To measure genomic distance among MATGs along with other publicly available *Treponema* genomes, amino acid usage, codon usage, and k-mer profile were determined and Euclidean distance was calculated by “comparem diss” commands implemented in CompareM program (40, 44, 45). The weighted Bray-Curtis approach was used to generate hierarchical cluster heatmaps based on Euclidean distance metric as a measure of distance between genomes (46)⁠. The clusters of orthologous groups (COG) functional annotation of coding sequences was performed using the Perl script CDD2COG.pl v.0.2 (47). The Manhattan distance and average clustering method built into the heatmap2 function of the gplots package of the R program were used to construct the heatmap to examine the distribution of genes in different COG functional categories (48, 49). The regions of differences (RODs) between reference *Treponema* genomes and MATGs were visualized by pairwise comparison using BLASTP searches in the CGView Comparison Tool (CCT) (50). MultiGeneBlast (51) was used to identify homologs of multigene clusters or operons between MATGs and publicly available *Treponema* genomes.

## RESULTS

### General genomic features of MATGs obtained from the elk hoof microbiome

Read statistics from shotgun metagenomes for the nine hoof samples analyzed in this study are shown in Table 1. Out of nine hoof sample metagenomes, only the two IDS skin scraping samples (20-01RF98 and 20-04LF98) collected from experimentally challenged elk yielded >10% bacterial reads, leading to successful construction of high-quality MATGs. In contrast, the metagenomes sequenced from biopsy samples yielded <5% bacterial reads and, as a result, these samples failed to yield high-quality MATGs for downstream analysis. The two skin scraping samples yielded a total of 10 high-quality MATGs with completeness of >90% and contamination of ˂3% (Table 2). Consequently, for the downstream analysis, we focused on these 10 MATGs constructed from skin scraping samples 20-01RF98 and 20-04LF98. The length of the 10 reconstructed MATGs ranged between 2.27 Mb (*Treponemataceae_*phy1_A17) and 2.82 Mb (*T. phagedenis*_A13) with the GC (guanine and cytosine) percentages ranging between 35% (*Treponemataceae*_phy1_A13) and 45.3% (*Treponema* sp._A13) (Table 2). The *Treponema* genomes available in the NCBI GenBank database (last accessed on 18 March 2023) were retrieved for comparative genomics analysis of the newly constructed MATGs. The lengths of the *Treponema* genomes downloaded from the NCBI GenBank database ranged from 1.13 (*Treponema paraluiscuniculi* Cuniculi A) to 4.05 Mb (*Treponema primitia* ZAS-2) and the GC percentage ranged from 36.8% (*Treponema pectinovorum* strain Marseille-1-CSURP6641) to 53.2% (*Treponema saccharophilum* DSM 2985) (Table S2).

**TABLE 1:** Read statistics of nine elk hoof sample metagenomes

Sample #	Type of sample	Raw read count (million)	Total microbial reads in millions (%)	Total host reads in millions (%)	Sample origin Reference
20-01RF98	Skin scraping	45.8	15.5 (33.9)	30.3 (66.1)	(3)
20-04LF98	Skin scraping	42.1	4.7 (11.2)	37.4 (88.8)	
16946RH	Biopsy	40.3	1.8 (4.6)	38.5 (95.4)	(2)
15288LH	Biopsy	43.6	1.5 (3.6)	42.0 (96.4)	
15074RF	Biopsy	45.2	1.8 (4.0)	43.3 (95.9)	
15074RH	Biopsy	40.8	1.7 (4.2)	39.1 (95.7)	
7559RH	Biopsy	40.2	1.5 (3.8)	38.7 (96.1)	
1978LH	Biopsy	41.7	1.7 (4.2)	39.9 (95.8)	
1972H1	Biopsy	43.4	1.7 (4.0)	41.7 (96)	

**TABLE 2:** General genomic features and quality metrics of 10 MATGs constructed from two elk hoof metagenomes

Sample	MATG name	MATG accession #	MATG size (bp)	GC (%)	CDS (%)	Genes (pseudo)	# of contigs/scaffolds	Completeness (%)	Contamination (%)
20-04LF98	*T. pedis*_A17	JARGEX000000000	2691800	37.2	88.8	2,463 (20)	117	98.36	0
20-01RF98	*T. pedis*_A13	JARGEW000000000	2636923	37.2	88.8	2,365 (21)	86	99.17	0
20-01RF98	*T. phagedenis*_A13	JARGEY000000000	2822893	40.1	81.5	2,372 (37)	126	97.58	1.61
20-04LF98	*T. phagedenis*_A17	JARGEZ000000000	2804377	40.3	81.	2,382 (37)	136	98.39	0.81
20-01RF98	*Treponema* sp._A13	JARGFA000000000	2323492	45.3	90.9	2,053 (24)	29	99.19	0
20-04LF98	*Treponema* sp._A17	JARGFB000000000	2385860	45.3	90.8	2,110 (27)	58	99.19	0
20-01RF98	*Treponemataceae _*phy1_A13	JARGFC000000000	2277200	35.0	84.8	2,077 (17)	103	97.9	0
20-04LF98	*Treponemataceae_*phy1_A17	JARGFE000000000	2092260	41.9	85.8	1,840 (22)	102	100	0
20-01RF98	*Treponemataceae_*phy2_A13	JARGFD000000000	2536991	35.4	83.9	2,337 (21)	176	100	0
20-04LF98	*Treponemataceae _*phy2 _A17	JARGFF000000000	2207218	42.0	85.7	1,982 (24)	130	99.19	1.61

^*a*^ GC, guanine (G) and cytosine (C); CDS, coding sequence.

### Genomic characterization of MATGs constructed from the elk hoof microbiome

**Fig 1:**
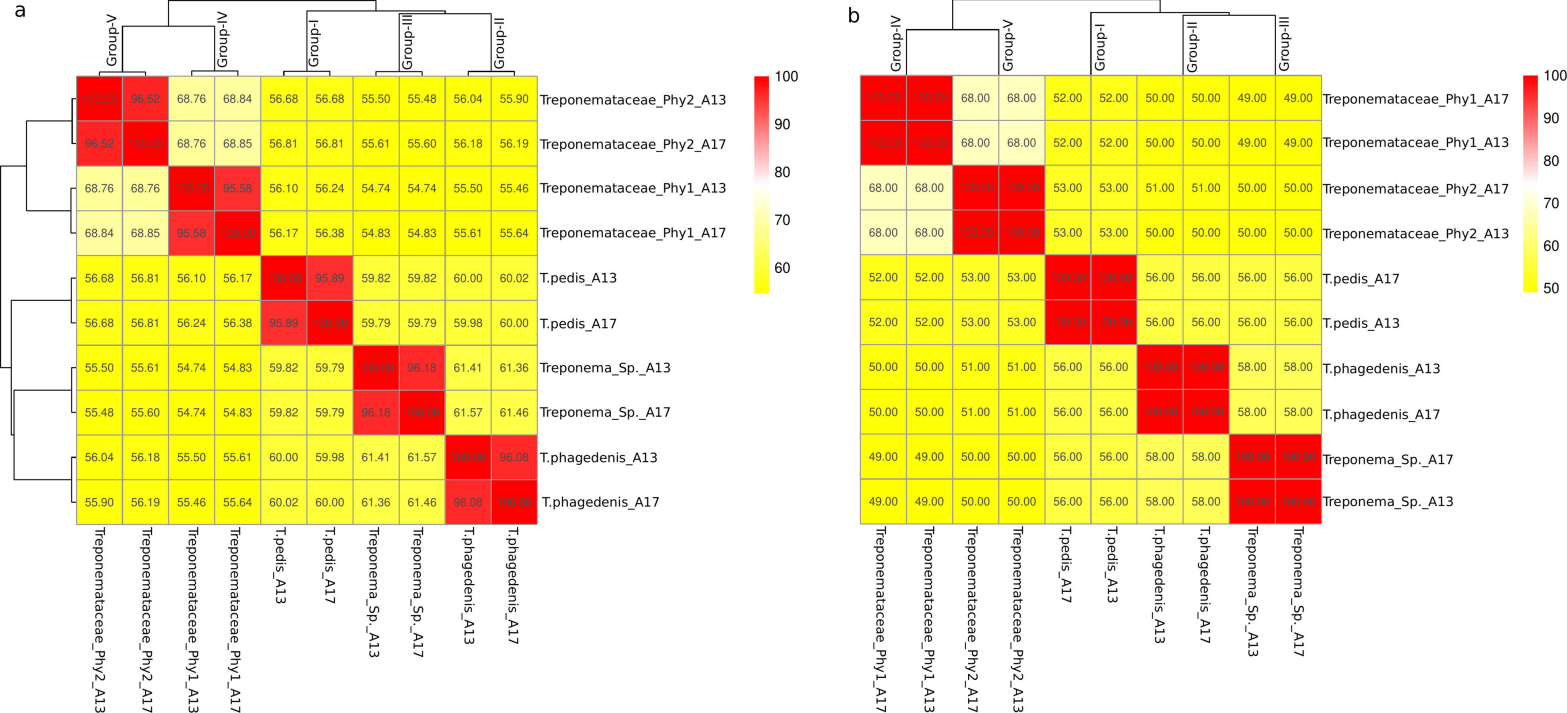
(**a**) ANI and (**b**) AAI matrix of 10 MATGs constructed from elk hoof samples. MATGs align into five groups based on ANI. The color gradient represents percent ANI (**a**) and AAI (**b**).

To examine the genetic relatedness between the 10 MATGs constructed in this study and publicly available *Treponema* genomes, the ANI, AAI, and POCP values were computed for all 10 MATGs. Based on the ANI and AAI analyses, the MATGs were grouped into five genogroups each with two MATGs representing the same species (Fig. 1a and b). When compared with the publicly available reference *Treponema* genomes, group I MATGs (*T. pedis_*A13 and *T. pedis*_A17) and group II MATGs (*T. phagedenis*_A13 and *T. phagedenis*_A17) displayed ANI values of >95% and AAI values of >96%, and thus were confidently assigned to the well-defined *Treponema* species group, i.e., *T. pedis* and *T. phagedenis*, respectively. However, group III MATGs (*Treponema* sp._A13 and *Treponema* sp._A17), group IV MATGs (*Treponemataceae*_phy1_A13 and *Treponemataceae*_phy1_A17), and group V MATGs (*Treponemataceae*_phy2_A13 and *Treponemataceae*_phy2_A17) displayed ANI values of <95% (Table S3) and AAI of <96% (Table S4). Taxonomic validation (41) could be achieved at the genus level for group III MATGs (POCP >50%); however, taxonomical validation could not be achieved at the genus level for group IV or group V MATGs (POCP <50%). The POCP analysis suggests that group III MATGs hold distinct taxonomic positions within the genus *Treponema*, and group IV and group V MATGs hold distinct taxonomic positions within the family *Treponemataceae* (Table S5) (41)⁠. Collectively, out of 10 MATGs identified in this study, two MATGs are identified as *T. pedis* (*T. pedis*_A13 and *T. pedis*_A17) and two are identified as *T. phagedenis* (*T. phagedenis*_A13 and *T. phagedenis_*A17). Out of the remaining six MATGs, two MATGs have been taxonomically classified as *Treponema* sp. (*Treponema* sp._A13 and *Treponema* sp._A17), exhibiting a POCP greater than 50%, indicating they belong to the same genus with an unknown species. On the contrary, the four MATGs recognized as *Treponemataceae* (*Treponemataceae*_phy1_A13, *Treponemataceae*_phy1_A17, *Treponemataceae*_phy2_A13, and *Treponemataceae*_phy2_A17) exhibit a POCP of less than 50% against reference *Treponema* genomes, leading to their taxonomic classification as a distinct genus within the *Treponemataceae* family.

### Core genome phylogeny

**Fig 2:**
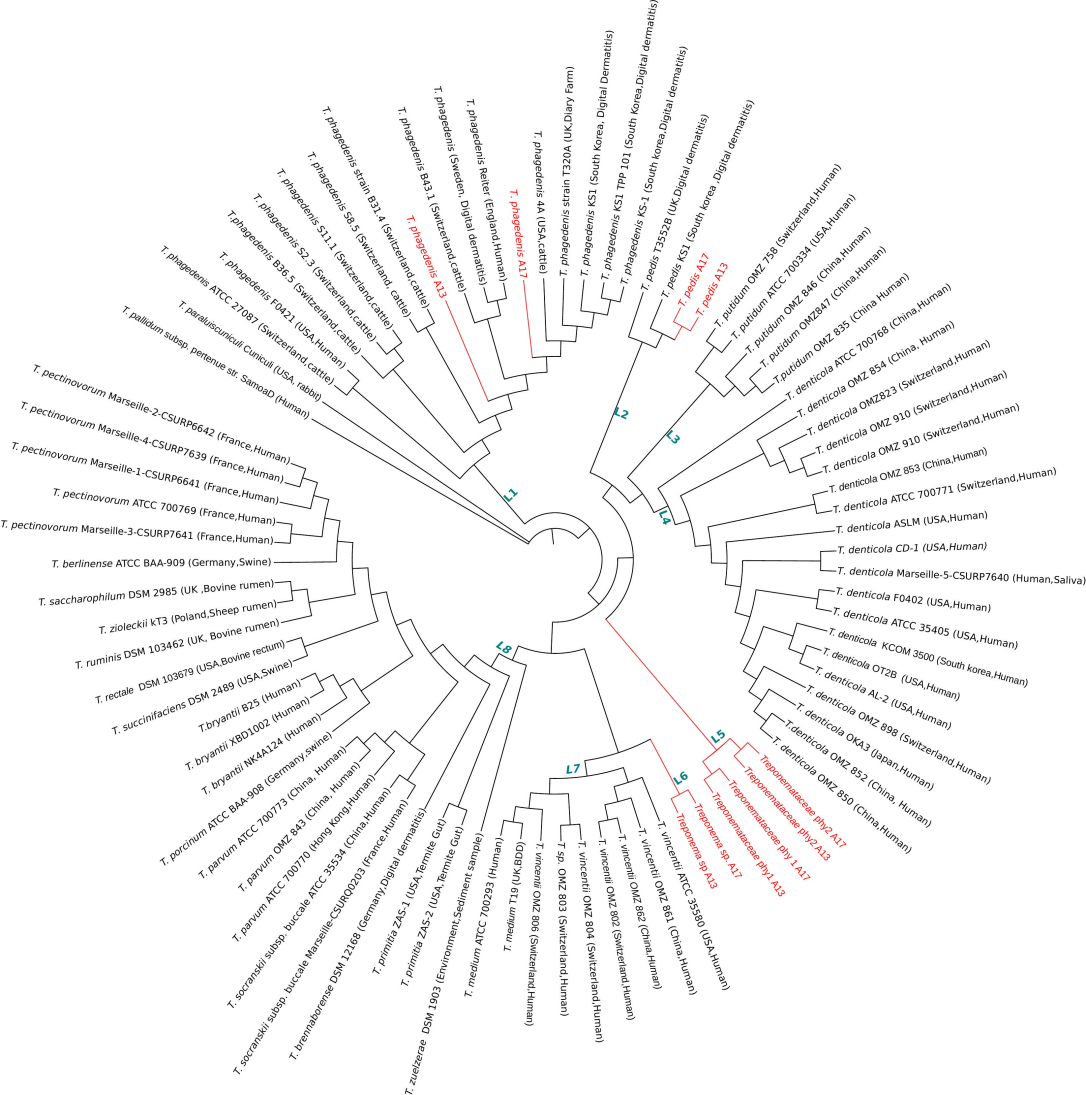
Maximum-likelihood core genome phylogenomic tree of the 10 MATGs constructed from elk hoof metagenomes and 75 publicly available *Treponema* genomes. This tree is derived from the concatenated alignment of 88 core genes, ensuring a robust phylogenetic reconstruction. Geographical locations and isolation sources of the genomes are annotated on tips of the tree. L1 to L8 represent distinct phylogenetic clades.

To determine the genomic relatedness of MATGs obtained from the elk hoof microbiome, we compared the core gene content of each MATG with 74 publicly available *Treponema* genomes, and one *T. pallidum* reference genome, all with quality criteria of >90% completeness and <5% contamination (Table S6) (33). Comparative genomics analysis identified 88 core genes that are conserved across all compared genomes. These 88 core genes were used to construct a non-recombinant single-copy core gene phylogenomic tree. The results show the existence of eight well-defined monophyletic clades, L1 to L8 (Fig. 2). Two *T. phagedenis* MATGs constructed from elk hoof metagenomes clustered within clade L1, which is composed of multiple *T. phagedenis* strains isolated and sequenced from diverse sources including human and cattle. Clade L1 forms multiple subclusters irrespective of the isolation source, suggesting significant genomic diversity within this clade. Similarly, two *T. pedis* MATGs constructed from elk hoof metagenomes cluster within clade L2 with two other *T. pedis* genomes isolated from cattle with digital dermatitis, one from the UK and one from South Korea. Interestingly, two elk hoof-derived MATGs of *Treponema* sp. (*Treponema* sp._A13 and *Treponema* sp._A17) formed an independent clade, L6, adjacent to *T. vincentii*/*T. medium* clade L7. All *T. vincentii* genomes within clade L7 originated from human sources, whereas the two *T. medium* genomes within this clade originated from cattle and a human source. Finally, the MATGs including *Treponemataceae*_phy1 and *Treponemataceae*_phy2 constituted a distinct basal clade L5 with the closest bordering branches including *T. pedis*, *T. putidum*, and *T. denticola* species.

### Profiling metabolic functions of MATGs derived from elk hooves

**Fig 3:**
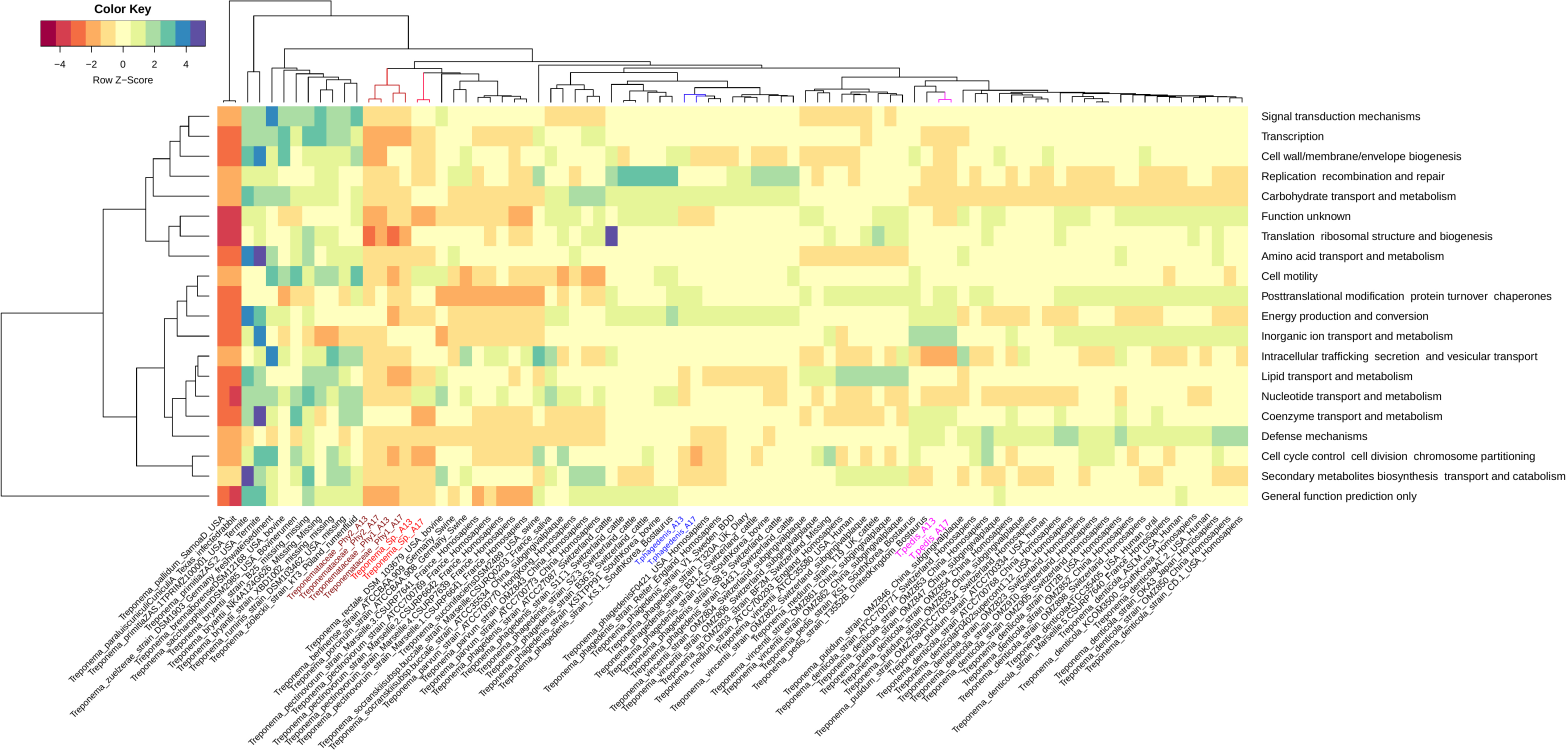
Heatmap depicting COG function profiles across *Treponema* genomes. Differentially enriched metabolic functions are presented for reference genomes (colored in black) and metagenome-assembled *Treponema* genomes from elk hooves (colored in red, blue, and pink). Raw z-score (scale range from −4 to +4) indicates abundance of genes associated with each functional category illustrated with brown (low abundance) and blue (high abundance) colors. The hierarchical clustering of genomes was performed using a weighted Bray-Curtis approach.

Metabolic profiling of bacterial genomes serves as an important adjunct in resolving taxonomic ambiguities of the newly discovered genomes (52–54). For metabolic profiling of MATGs constructed in this study, we determined the COG metabolic functions of all gene content of each MATG and compared them with 75 publicly available *Treponema* genomes. Hierarchical clustering showed that hoof-derived MATGs constructed in this study were clustered into COG functional groups (Fig. 3) that aligned with the genogroup clusters (Fig. 2) based on the core genome phylogeny. MATGs possessed more than 1,300 functionally active genes (Fig. 3). As expected, *T. pallidum* (794 functional genes) and *T. paraluiscuniculi* (797 functional genes) harbored fewer functional genes, indicating overall diminished metabolic capability of these organisms when compared with the other *Treponema* species. Among all the elk hoof-derived MATGs, *Treponemataceae*_phy1, *Treponemataceae*_phy2, and *Treponema* sp. showed significantly lower COG functional enrichment relative to other non-*Treponema pallidum* genomes (Fig. 3), indicating metabolic distinction of these MATGs.

### Comparative genomics and cluster analysis of MATGs

**Fig 4:**
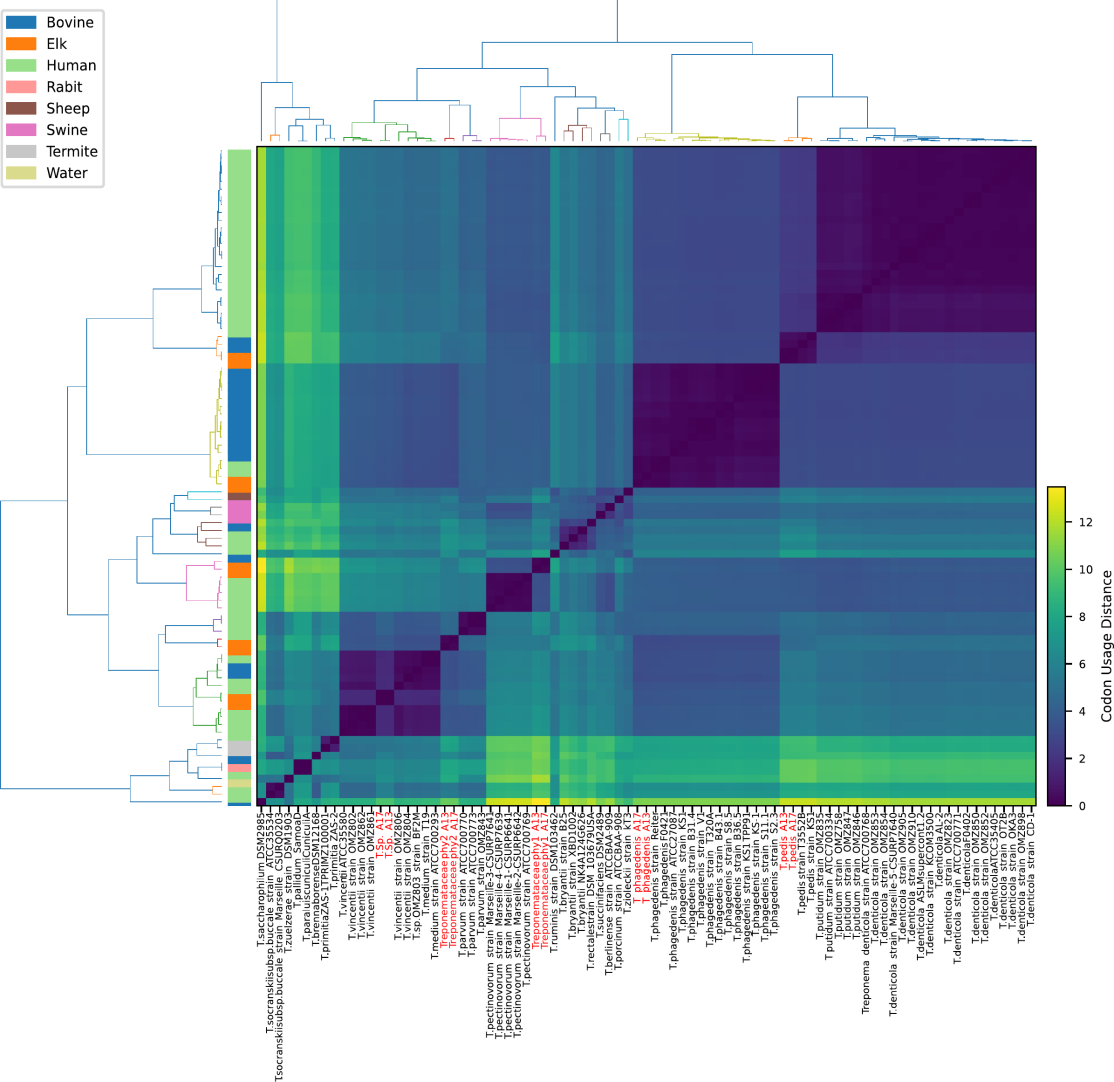
Heatmap representing codon usage distance among *Treponema* genomes and MATGs from elk hooves. The hierarchical clustering of strains was conducted using the average linkage method and the Bray-Curtis dissimilarity metric.

**Fig 5:**
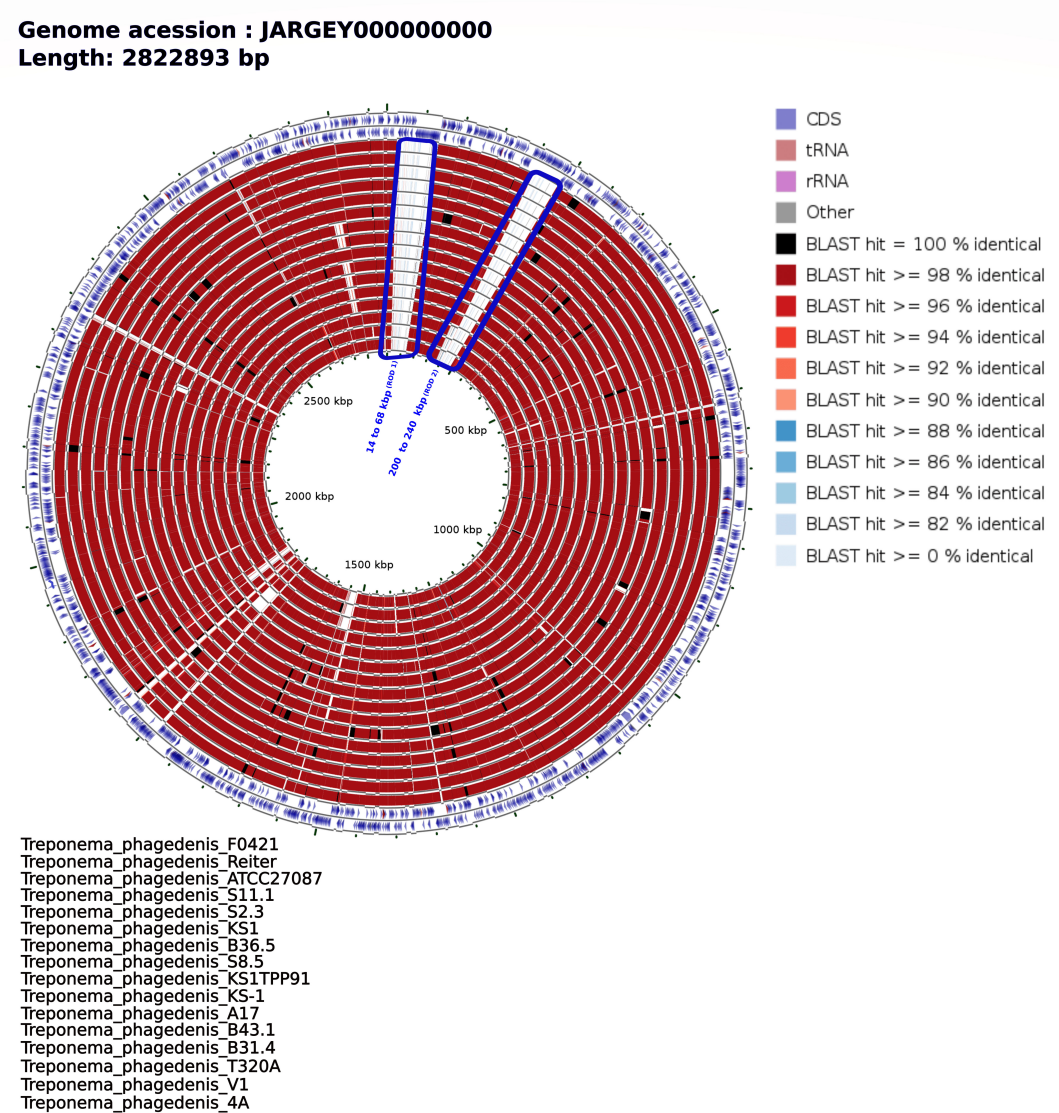
Genome-wide comparison of *Treponema phagedenis* strains. Circular map representing *T. phagedenis*_A13 genome-wide comparison with other publicly available *T. phagedenis* strains. The percentage similarity is represented by different color codes from outer circle to inner as follows: genome size, genes on the forward strand, genes on the reverse strand, tRNA, and rRNA. The outermost rings (blue) show two strands of *T. phagedenis*_A13 MATG. The inner 16 rings (red) show the BLAST sequence similarity comparisons between the *T. phagedenis*_A13 and 16 *T. phagedenis* genomes, including *T. phagedenis*_A17 MATG (ring 11), sequentially from inner to outer circle as referenced in the bottom left of the figure.

**Fig 6:**
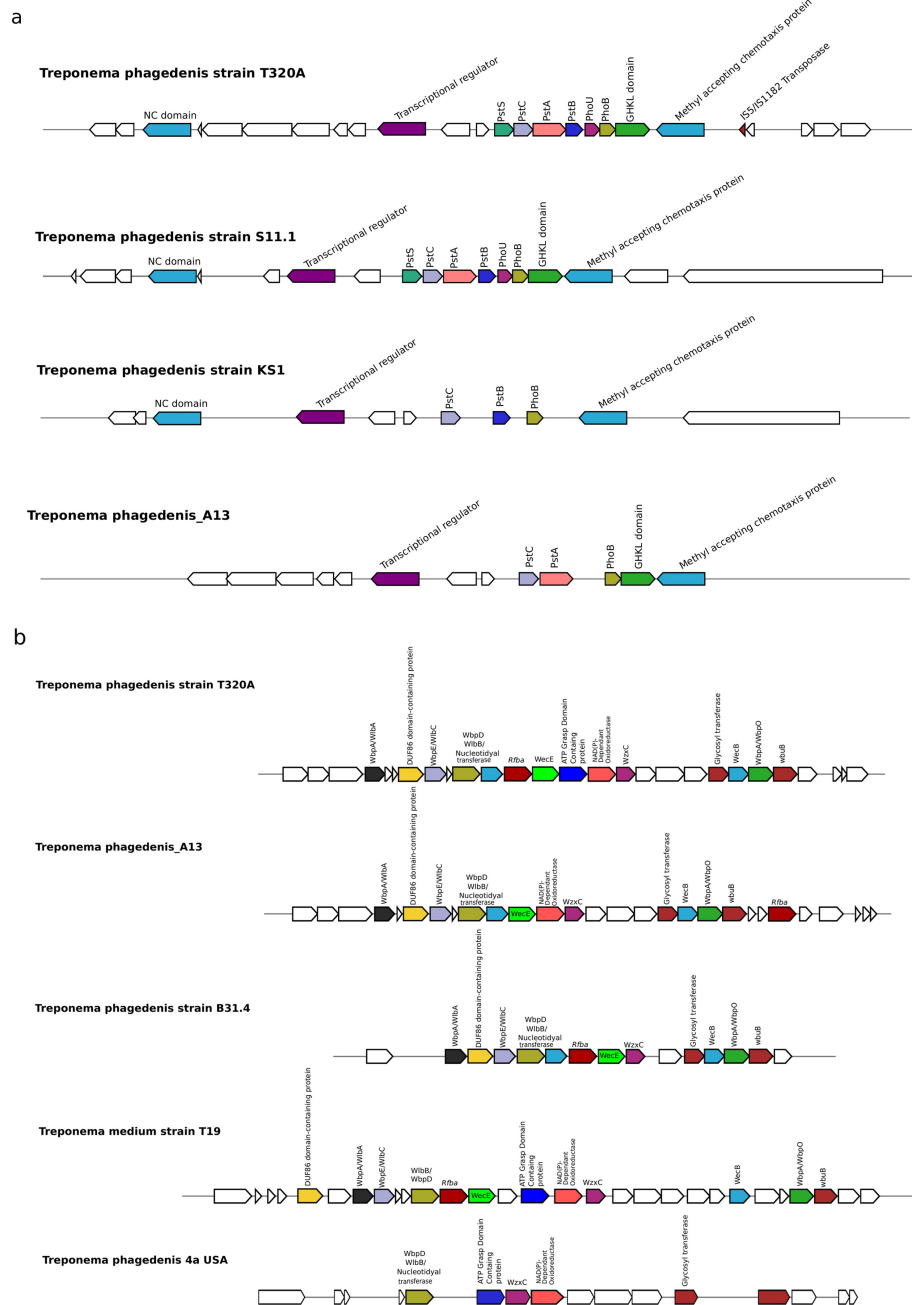
Comparison of *T. phagedenis* T320A genome with other *T. phagedenis* strains for the presence of two bovine-specific gene clusters. (**a**) Phosphate utilization gene cluster. (**b**) Gene cluster associated with outer membrane production.

To further explore genomic distances between *Treponema* genomes and newly constructed MATGs, we compared codon usage, amino acid usage, and k-mer frequencies (44, 52). The clustering pattern among MATGs based on codon usage (Fig. 4), amino acid usage (Fig. S1), and k-mer frequency analysis (Fig. S2) corroborated with the ANI and AAI analyses results. To identify major genomic differences between elk hoof-derived MATGs and other closely related *Treponema* genomes, we compared publicly available *Treponema* genomes sharing the same clade with the elk hoof-derived MATGs using the CGView Comparison Tool (Fig. 2). This analysis identified a few unique genomic regions, referred to as RODs, in elk hoof-derived MATGs. For instance, comparison of *T. phagedenis* MATGs with the *T. phagedenis* genomes within clade L2 identified two RODs ranging in size from 14 kbp to 68 kbp (*T. phagedenis*_A13-ROD 1) and 200 kbp to 240 kbp (*T. phagedenis*_A13-ROD 2). These RODs are present in *T. phagedenis*_A13 MATG, but not in *T. phagedenis*_A17 MATG (Fig. 5). Noteworthy is that these two MATGs (*T. phagedenis*_A13 and *T. phagedenis*_A17) were constructed from the same sample and showed only 2% to 6% dissimilarity at the nucleotide level. *T. phagedenis*_A13-ROD 1 contains approximately 53 genes, whereas *T. phagedenis*_A13-ROD 2 contains approximately 18 genes that are exclusive to the elk hoof-derived *T. phagedenis*_A13 MATG. The majority of the genes within these RODs encode proteins associated with flagellar structures, transport functions, and glycosyltransferase activity (Table S7). These RODs are distinct from recently reported bovine-specific gene clusters associated within the *T. phagedenis* genome (14), one encoding machinery for outer membrane protein (OMP) synthesis and another constituting two polycistronic operons for phosphate utilization systems (Fig. 6). The analysis of the two bovine-specific gene clusters revealed that the elk hoof-derived *T. phagedenis*_A13 MATG possessed the OMP gene cluster while lacking a complete phosphate utilization gene cluster (14). Conversely, both gene clusters are absent in the *T. phagedenis*_A17 MATG. It is worth noting that both gene clusters were also missing or incomplete in *T. phagedenis* 4A, a bovine isolate from the USA (13). Therefore, although *T. phagedenis*_A13 MATG and *T. phagedenis*_A17 MATG cluster with *T. phagedenis* isolated from cattle (Fig. 2), carriage of two distinct RODs within *T. phagedenis*_A13 MATG shows that this MATG is genetically distinct from *T. phagedenis*_A17 MATG and all other *T. phagedenis* isolated to date from domestic cattle.

**Fig 7:**
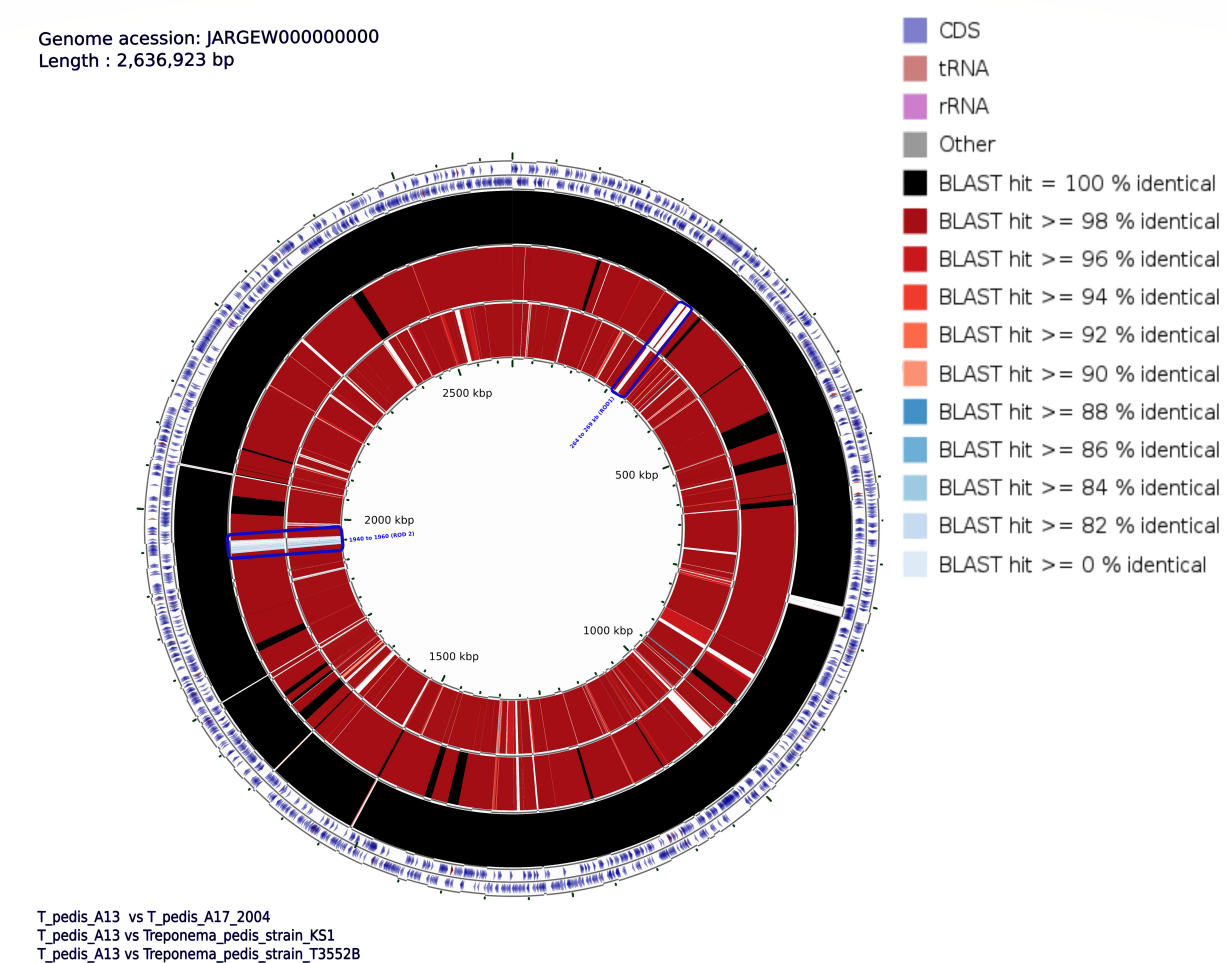
Circular map representing *Treponema pedis*_A13 genome-wide comparison with other reference *T. pedis* strains. The percentage similarity is represented by different color codes. The information is read from outer circle to inner as follows: genome size, genes on the forward strand, genes on the reverse strand, tRNA, and rRNA. The next three rings show the sequence similarity by BLAST comparisons between the *T. pedis*_A13 and three other *T. pedis* genomes, sequentially from inner to outer circle as referenced in the bottom left of figure.

The comparison of two elk hoof-derived *T. pedis* MATGs with *T. pedis* genomes isolated from cattle also identified two RODs ranging from 264 to 269 kbp (*T. pedis*-ROD 1) and 1,940 to 1,960 kbp (*T. pedis*-ROD 2) that are exclusive to the elk hoof-derived *T. pedis* MATGs (Fig. 7). *T. pedis*_A13-ROD 1 contains seven genes, while *T. pedis*_A13-ROD 2 contains 19 genes that are specific to the *T. pedis* MATGs. The unique genes identified in these elk hoof-derived MATGs encode diverse metabolic functions, without an over representation of genes associated with any specific functional group (Table S7). *T. pedis* MATGs (*T. pedis*_A13 and *T. pedis*_A17) exhibited 100% genome-wide similarity despite being reconstructed from two different samples (Fig. 7).

**Fig 8:**
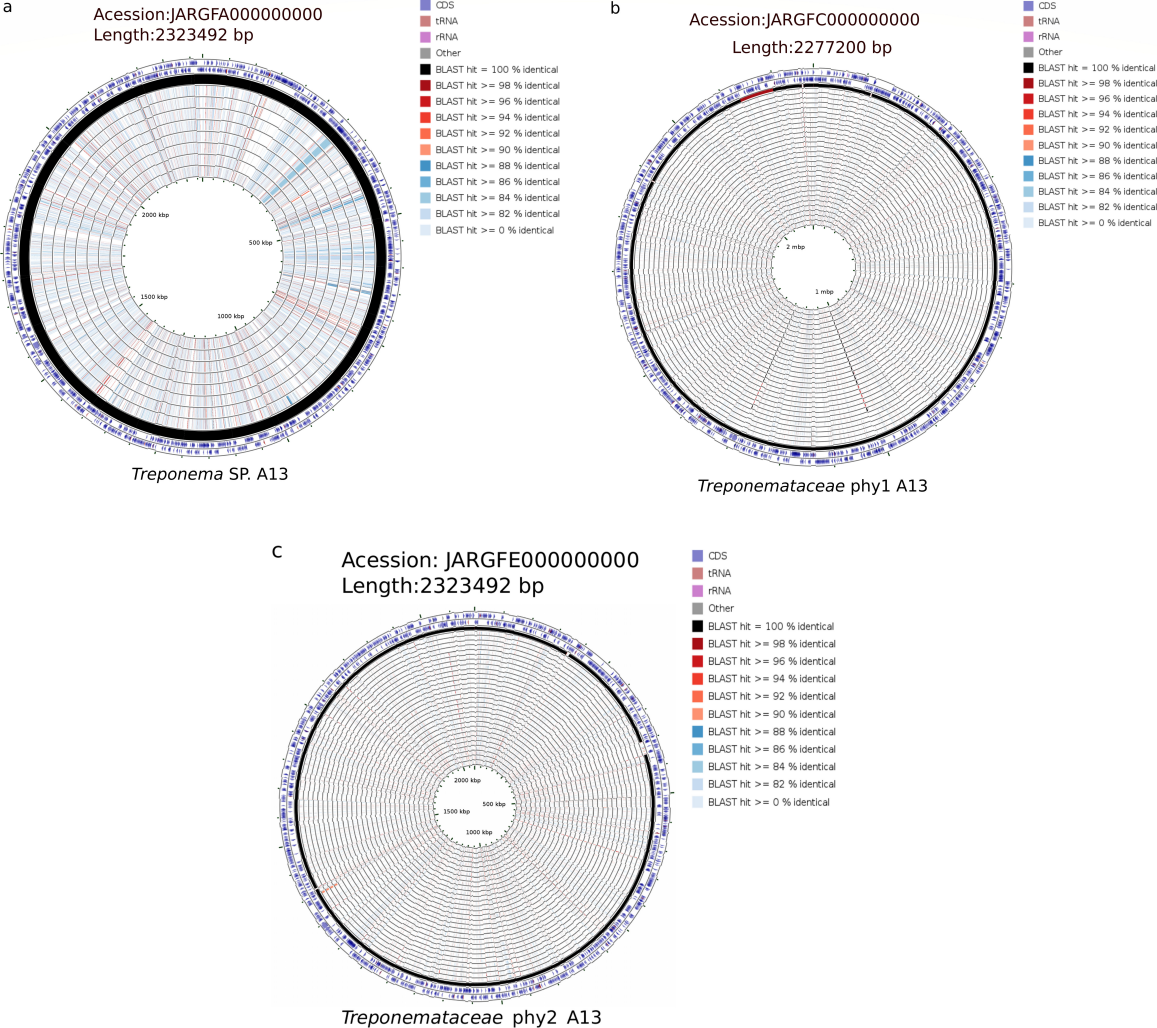
Circular plot of *Treponema* sp._A13 (**a**), *Treponemataceae*_phy1_A13 (**b**), and *Treponemataceae*_phy2_A13 (**c**) MATGs compared with other *Treponema* genomes via CCT. The details of each concentric ring are described in File S1.

Contrary to the above observations on *T. phagedenis* and *T. pedis* MATGs, the elk hoof-derived MATGs within the other three taxonomic genogroups (*Treponema* sp., *Treponemataceae*_phy1, and *Treponemataceae*_phy2) showed no significant similarity to any of the genomes with their closest relatives within clades L7 and L4 (Fig. 8). These MATGs showed genome-wide dissimilarity of ≥80% at the nucleotide level when compared with publicly available *Treponema* genomes within clade L4, suggesting their taxonomic novelty within the *Treponemataceae* family.

### Taxonomic analysis for identification of MATG-specific signatures in biopsy samples

**Fig 9:**
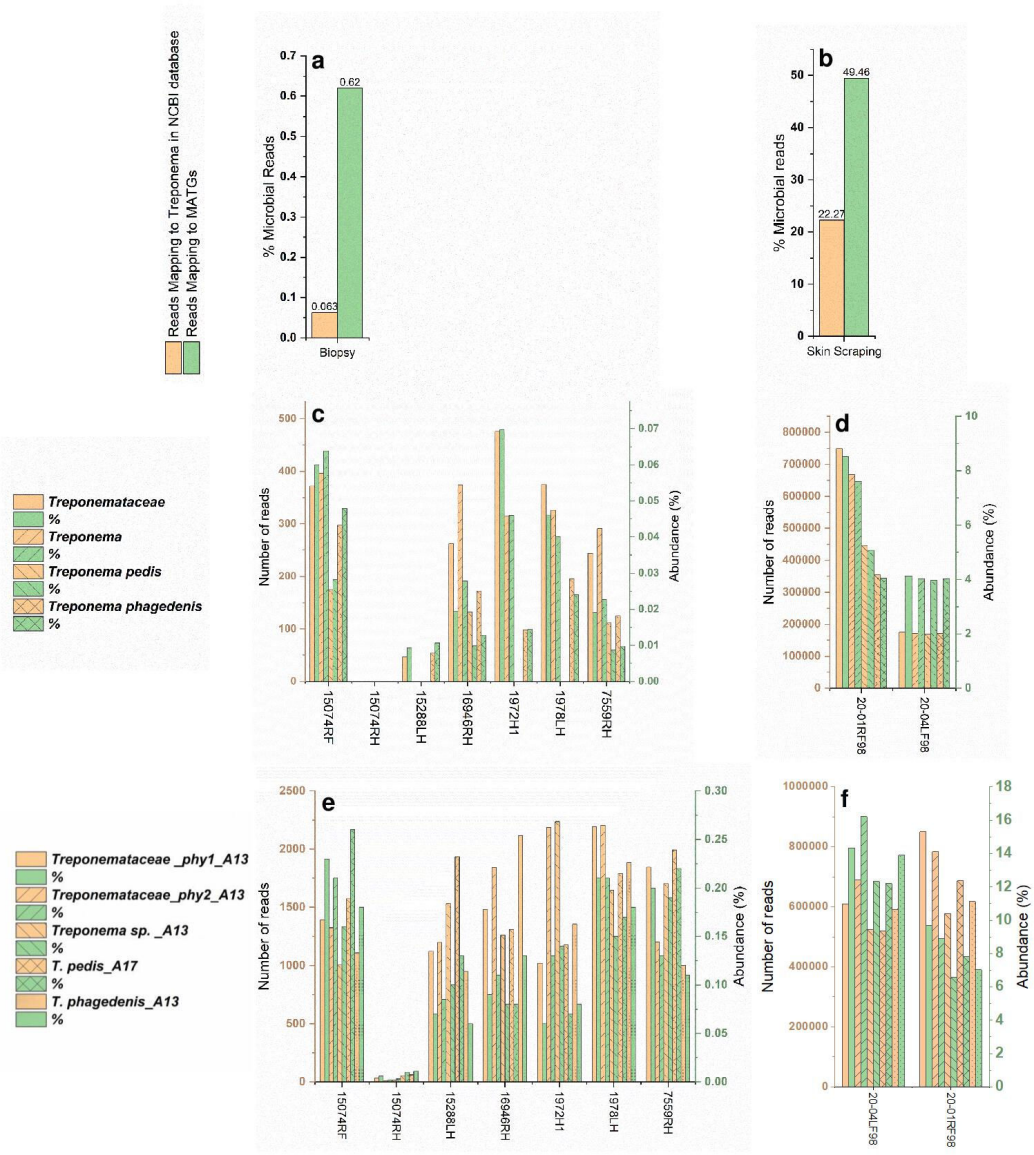
Relative abundance of *Treponemataceae* members in biopsy (left panel) and skin scraping (right panel) samples obtained via taxonomic analysis of reads using Kraken 2 pipeline (**c and d**) and by back mapping of reads to MATGs constructed in this study (**e and f**). The top panels show the total percent microbial reads detected in biopsy (**a**) and skin scraping (**b**) samples using Kraken 2 pipeline and back mapping to MATGs.

Due to the relatively low numbers of microbial reads obtained from biopsy samples when compared with the skin scraping samples (Table 1), none of the binning approaches could successfully reconstruct MATGs from the biopsy samples. Consequently, to determine if biopsy samples carried any known or novel *Treponema* signatures, we employed an alternative approach including taxonomic analysis using the Kraken 2 pipeline (30), and back mapping reads to the MATGs constructed in this study (Fig. 9). With the Kraken 2 pipeline, each read was assigned to NCBI taxonomic ID, using the lowest common ancestor-based method, whereas with back mapping, each read was assigned a MATG-specific taxonomic ID. For comparison, skin scraping samples were included in this analysis. The results show that the percent abundance of microbial reads in biopsy samples mapping to known *Treponema* signatures in the current NCBI GenBank database (0.063%) was 10 times lower when compared with the microbial reads from the biopsy samples mapping to the MATGs (0.62%) constructed in this study (Fig. 9a). Similarly, a lower proportion of microbial reads from skin scrapings mapped to known *Treponema* signatures in the NCBI GenBank database (22%) when compared with reads mapped to MATGs (49%) (Fig. 9b). More specifically, results of analysis using Kraken 2 pipeline revealed that six biopsy samples carried a low abundance of reads mapping to *Treponemataceae* (0.002% to 0.05%) and *T. phagedenis* (0.003% to 0.04), five samples showed a low abundance of reads mapping to *Treponema* (0.02% to 0.06%), three samples showed a low abundance of reads mapping to *T. pedis* (0.01% to 0.03%), and one sample (15074RH) failed to yield any taxonomic ID (Fig. 9c). In contrast, a higher abundance of reads (1.76% to 5.11%) from each skin scraping mapped to these four taxa (Fig. 9d). Back mapping of microbial reads from biopsy samples revealed varying proportions of reads from all seven (100%) biopsy samples mapped to five representative MATGs identified in this study including *Treponemataceae_*phy1_A13 (0.01% to 0.14%), *Treponemataceae_*phy2_A13 (0.01% to 0.16%), *Treponema* sp._A13 (0.001% to 0.14%), *T. pedis*_A17 (0.003% to 0.15%), and *T. phagedenis*_A13 (0.003% to 0.73%) (Fig. 9e). Similar results were obtained when microbial reads from skin scrapings were back mapped to MATGs (Fig. 9f). Given that the current NCBI GenBank database does not include the genomes of novel MATGs constructed in this study, taxonomic analysis is not expected to detect any MATG-specific signatures. These results show that the biopsy samples contained both the reads specific to known *Treponema* signatures in the current database and reads that are unique to MATGs. Furthermore, the detection of novel MATG-specific signatures in biopsy samples collected from free-ranging elk with naturally occurring TAHD and from the skin scrapings collected from captive elk early during the experimentally induced disease provides robust evidence that the novel MATGs identified in this study are likely associated with pododermatitis in free-ranging elk.

## DISCUSSION

Identifying and characterizing putative pathogens is crucial to support the development of sensitive and specific assays and for etiological and epidemiological investigations for emerging diseases such as pododermatitis in elk. *Treponema* is identified as a consistent and dominant bacterial group in digital dermatitis in livestock and TAHD in elk through immunohistochemistry, PCR, and V3-V4 amplicon sequencing (2, 55). Although research on digital dermatitis in domestic livestock has yielded a limited number of complete genomes of a few culturable *Treponema* spp., the lack of publicly available *Treponema* genomes derived from pododermatitis lesions in elk leaves a significant gap in our knowledge of TAHD’s etiology and epidemiology.

The comprehensive approach employed in this study, including the reconstruction of novel MATGs from the shotgun metagenome of clinical samples and comparative genomics analysis, broadens our knowledge by capturing a wider range of yet uncultured *Treponema* genomes linked to pododermatitis in elk. Among the nine elk hoof samples examined in this study, the bioinformatics pipeline effectively constructed novel MATGs from two skin scraping samples (20-01RF98 and 20-04LF98) from live elk. Given that the tissue biopsies used in this study originated from a convenience sampling of elk mortalities from the field, inconsistencies in the sample freshness, collection, storage, and transportation may have resulted in poor gDNA quality leading to low bacterial read outputs for downstream shotgun metagenomic assemblies. Furthermore, biopsies are naturally highly enriched with host reads as sampling includes deeper tissues with higher host DNA biomass, relative to skin scraping, potentially masking the microbial read outputs and leading to decreased sensitivity for microbial detection. Despite these challenges hindering our ability to reconstruct MATGs from biopsy samples effectively, our alternative approach to utilize a combination of taxonomic analysis of reads from each biopsy sample using the Kraken 2 pipeline and back mapping of biopsy reads against recovered MATGs allowed successful detection and determination of the proportion of novel MATG-specific signatures in all biopsy samples tested. These results suggest that the novel MATGs identified in this study are associated with pododermatitis. The availability of this new genomic information in the publicly available genomic database will facilitate follow-up investigations into the etiology and epidemiology of pododermatitis in elk.

While MATGs constructed in this study were from samples collected from captive experimental elk, the study design and results of the previously reported captive elk study provide confidence that the source of MATGs was from lesions induced by challenge with inoculum prepared from TAHD-infected feet of free-ranging elk (3). MATGs were associated with the development of lesions in experimentally challenged elk that were indistinguishable from those in free-ranging elk (3). This finding combined with the detection of MATGs in biopsies from free-ranging elk by back mapping conducted in this study confirms that these MATGs are present not just in captive experimental elk, but in free-ranging elk. Thus, the successful reconstruction of MATGs from skin scraping samples, which may contain relatively less host biomass and serve as a rich source of microbial signatures, suggests that skin scrapings may potentially serve as more representative samples than biopsies for investigating pododermatitis in elk.

The bioinformatics pipeline employed in this study effectively constructed MATGs with robust evidence of their taxonomic rank through core genome phylogeny, ANI, AAI, and POCP analysis. Notably, four MATGs (group I and group II) provided species-level resolution, and six MATGs (group III, group IV, and group V) provided identification of novel genomospecies within the *Treponemataceae* family (Fig. 1). The three unresolved taxa, namely *Treponema* sp., *Treponemataceae*_phy1, and *Treponemataceae*_phy2, can be regarded as new additions to the treponemal group associated with TAHD. Hierarchical clustering based on amino acid usage, codon usage, and k-mer frequencies reinforced the genomic differences between currently known *Treponema* species and novel MATGs and supports distinct species groups identified in this study. The discovery of novel *Treponema* MATGs suggests the continued expansion of members within the family *Treponemataceae* and increased genetic diversity within the phylum Spirochaetota (56, 57). Our bioinformatics pipeline does not yield full-length 16S rDNA sequences. Thus, it remains unknown if any of the MATGs constructed in this study indeed represent PT19 as reported previously using 16S amplicon sequencing (2). Follow-up studies including reconstruction of novel MATGs from additional samples from free-ranging elk will further strengthen the association and distribution of these MATGs with pododermatitis in wild populations.

In addition to the discovery of novel MATGs, our findings corroborate the significant genetic diversity reported in *T. phagedenis*-like treponemes linked to BDD (58, 59). The data show distant phylogenetic placement of the two *T. phagedenis* MATGs based on core genome content (Fig. 2). For instance, *T. phagedenis*-ROD 1 and *T. phagedenis*-ROD 2 (Fig. 5) represent *T. phagedenis*_A13 MATG-specific features containing genes encoding a flagellar protein, transporter protein, glycosyltransferase, and several other transferase enzymes. These ROD 1- and ROD 2-specific genes might play a crucial role in conferring motility (60), environmental fitness (61), and lipopolysaccharide diversity (62) to the *T. phagedenis*_A13 strain. These findings suggest that *T. phagedenis*_A13 MATG is likely genetically distinct from *T. phagedenis_*A17 MATG and other previously reported *T. phagedenis* strains (Table S7). A recent study reported that bovine strains of *T. phagedenis* carry two bovine digital dermatitis-specific gene clusters (14). Of the two gene clusters, the OMP gene cluster was absent in *T. phagedenis*_A17 MATG and present in *T. phagedenis*_A13 MATG. Interestingly, *T. phagedenis* 4A, the only bovine isolate from the USA available for comparison, also lacks both gene clusters. It is important to note that the majority of *T. phagedenis* genomes analyzed in this study originated from sources outside the USA. As a result, it is unclear whether *T. phagedenis*_A17 and *T. phagedenis*_A13 MATGs constructed in this study are unique to elk or also occur in livestock in the USA. Variability in genetic clusters across *T. phagedenis* clones from different geographic regions emphasizes the need for further investigation into elk- and livestock-derived *Treponema* genomes to effectively capture the genetic diversity and discern the host and geographic distribution of these potential pathogens.

The genome-wide BLAST analysis revealed the two elk hoof-derived *T. pedis* MATGs constructed in this study share a 100% sequence identity across the genome (Fig. 7). However, the presence of two elk hoof-derived *T. pedis* MATG-specific genomic RODs (*T. pedis*-ROD 1 and *T. pedis*-ROD 2) raises a possibility that elk hoof-derived *T. pedis* is genetically distinct from other *T. pedis* strains. The unique regions in *T. pedis* and *T. phagedenis* MATGs have likely been acquired through horizontal gene transfer occurring throughout the lineage evolution of *Treponema* genomes (63). The clustering of *T. medium* and *T. vincentii* clade L5 (Fig. 2) also corroborates with previously reported close clustering of these two species (8, 64)⁠. However, sub-clustering of *Treponema* sp. MATGs in clade L6 alongside *T. medium* and *T. vincentii* clade L5 implies that the *Treponema* sp. MATG potentially represents a novel addition to the existing cluster 4 treponeme phylogenetic group as reported by Yano et al. (64).

Microbial minimalism, a process of genome reduction in bacterial pathogens, limits the metabolic capabilities of these pathogens to better adapt to the host environment (65). Within the *Treponema* genus, the differentiation of *T. pallidum* and *T. paraluiscuniculi* is marked by their limited genome sizes and reduced metabolic activities (Fig. 3). In *T. pallidum* and *T. paraluiscuniculi*, highly reduced metabolic capacity is associated with the ongoing genome reduction process and loss of metabolic and biosynthetic pathways (66, 67). Interestingly, in our analysis, strains from the same *Treponema* species were aggregated in a single group, indicating that each member of the *Treponema* genus has a distinctive metabolic functional profile at the species level (Fig. 3). Among the elk hoof-derived MATGs, *Treponemataceae*_phy1, *Treponemataceae*_phy2, and *Treponema* sp. MATGs showed significantly lower COG functional enrichment relative to other non-*Treponema pallidum* genomes (Fig. 3). For instance, *Treponema* sp. MATGs carry an average of 58 and 88 functional genes associated with nucleotide transport metabolism and cell motility, respectively. In contrast, *Treponemataceae* MATGs carry 39 and 57 genes for these two respective functions. Overall, the novel MATGs derived from elk hooves show a considerably reduced metabolic capacity (Fig. 3). The observed reduced metabolic capacity in elk hoof-derived *Treponemataceae* MATGs aligns with metabolically distinct strains within the *Treponemataceae* family associated with vertebrates (68). The diminished metabolic capability of these elk hoof-derived MATGs is not surprising, given the relatively small genome size, host dependency, and fastidious nature (66, 67), and likely contributes to challenges in successful culturing and increased host dependence and adaptation (69). Thus, the culture-independent *in silico* approach for the identification and characterization of MAGs from clinical samples employed in this study could serve as a valuable tool for continued investigations into the etiology and epidemiology of pododermatitis in elk with potential translational applications in investigations of other diseases where etiological agents remain obscure.

### Conclusion

This study addresses the critical need for comprehensive identification of *Treponema* species associated with pododermatitis in elk. By employing a robust bioinformatic pipeline for metagenome reconstruction and taxonomic classification of novel *Treponema* from clinical samples, this study enhances our knowledge of genomic diversity within the *Treponemataceae* family. The study successfully uncovered novel treponemal groups within the *Treponemataceae* family, emphasizing the need to expand the data set of *Treponema* genomes linked to elk pododermatitis. The application of this bioinformatic pipeline to hoof samples from cattle and sheep holds promise for yielding similar insights into *Treponema* associated with digital dermatitis in livestock. Observations on the reduced metabolic capacity in elk hoof-derived *Treponemataceae* MATGs offer valuable insights into potential fastidiousness, which presents challenges in successful culturing, and host dependency, consistent with patterns observed in other *Treponema* species associated with vertebrates. In summary, this study identifies novel genomospecies of *Treponema* associated with pododermatitis in elk, thereby enhancing our understanding of the etiology and epidemiology of this disease. It underscores the ongoing need for research to expand the data set of *Treponema* genomes, which is essential for capturing genetic diversity and unraveling host and geographic distribution of these pathogens. These efforts are crucial for combating emerging diseases such as pododermatitis in both wildlife and livestock populations.

## Data Availability

The raw shotgun sequence reads are available in the NCBI-SRA database under the following accession numbers: SRR23724925 and SRR23724921. All 10 metagenome-assembled *Treponema* genomes are available in NCBI GenBank under the following accession numbers: JARGEX000000000 (T. pedis_A17), JARGEW000000000 (T. pedis_A13), JARGEY000000000 (T. phagedenis_A13), JARGEZ000000000 (T. phagedenis_A17), JARGFA000000000 (Treponema sp._A13), JARGFB000000000 (Treponema sp._A17), JARGFC000000000 (Treponemataceae_phy1_A13), JARGFE000000000 (Treponemataceae_phy1_A17), JARGFD000000000 (Treponemataceae_phy2_A13), and JARGFF000000000 (Treponemataceae_phy2_A17).

## SUPPLEMENTAL MATERIAL

Figure S1 aem.00105-24-s0001.svg Heatmap showing amino acid usage distance among Treponema genomes.

Figure S2 aem.00105-24-s0002.svg Heatmap showing k-mer frequency profile among Treponema genomes.

Supplemental legends aem.00105-24-s0003.docx Legends for supplemental tables and figures; extended description of Fig. 8.

Table S1 aem.00105-24-s0004.xlsx Sample metadata information.

Table S2 aem.00105-24-s0005.xlsx Genomic features of reference genome.

Table S3 aem.00105-24-s0006.xlsx ANI analysis.

Table S4 aem.00105-24-s0007.xlsx Average amino acid identity (AAI) analysis.

Table S5 aem.00105-24-s0008.xlsx Comparison of POCP.

Table S6 aem.00105-24-s0009.xlsx Assessment of completeness and contamination.

Table S7 aem.00105-24-s0010.xlsx List of unique genes.

ASM does not own the copyrights to Supplemental Material that may be linked to, or accessed through, an article. The authors have granted ASM a non-exclusive, world-wide license to publish the Supplemental Material files. Please contact the corresponding author directly for reuse.
