# Supplementary material for: Discovery of novel treponemes associated with pododermatitis in elk (Cervus canadensis)
Source: Appl Environ Microbiol. 2024 May 14;90(6):e00105-24. doi: 10.1128/aem.00105-24 (PMC11218636; doi:10.1128/aem.00105-24)
Supplement: Supplemental legends — Legends for supplemental tables and figures; extended description of Fig. 8. [file aem.00105-24-s0003.docx]

**Table List**

Supplementary Table 1: Comprehensive overview of nine samples collected from elk hooves.

Supplementary Table 2: Genome statistics and metadata for isolate *Treponema* genomes retrieved from the NCBI Database.

Supplementary Table 3: ANI analysis among publicly available *Treponema* genome and MATGs. ANI values of >95% represent same species; ANI of <95% are distinct species.

Supplementary Table 4: Average amino acid identity (AAI) analysis among *Treponema* genomes and MATGs. AAI values of >96% represent same species; ANIs of <96% are distinct species.

Supplementary Table-5: Comparison of POCP (Percentage of Conserved Proteins) values between MATGs and reference *Treponema* genomes to assess the genus boundary. POCP value >50 % indicates two genomes belongs to the same genus.

Supplementary Table 6: Assessment of completeness and contamination for reference *Treponema* genomes obtained from the NCBI Database.

Supplementary Table 7: List of genes unique to *T. pedis A13* and *T. phagedenis A13* MATG.

**Supplementary figure:**

Supplement Figure S1:

Heatmap showing the amino acid usage distance among Treponema genomes. The hierarchical clustering of the strains was based on average linkage method and Bray-Curtis dissimilarity metric.

Supplement Figure S2:

Heatmap showing k-mer frequency profile among Treponema genomes. The hierarchical clustering was based on average linkage method and Bray-Curtis dissimilarity metric.

**Figure 8**

Figure 8a:

Circular plot of Treponema Sp A13 genome compared with additional available Treponema genomes via CGView comparison tool (CCT). CDS in forward and reverse directions for Treponema Sp A13 genome are indicated in the outer rings, and BLAST-based comparisons to additional reference Treponema genomes are presented as inner rings in order of highest to lowest similarity to Treponema Sp A13 2001 genome. The innermost comparison ring begins with Treponema Sp A17, Treponema vincentii strain OMZ806, Treponema medium strain ATCC700293, Treponema vincentii strain OMZ802, Treponema medium strain T19, Treponema vincentii strain OMZ861, Treponema vincentiiATCC35580, Treponema vincentii strain OMZ862 and Treponema vincentii strain OMZ804.

Figure 8b:

Circular plot of Treponemataceae Phy1 A13 genome compared with additional available Treponema genomes via CGView comparison tool (CCT). CDS in forward and reverse directions for Treponemataceae Phy1 A13 2001 genome are indicated in the outer rings, and BLAST-based comparisons to additional reference Treponema genomes are presented as inner rings in order of highest to lowest similarity to Treponemataceae Phy1 A13 genome. The innermost comparison ring begins with Treponematace_phy1_A17, Treponema pedis strain KS1, Treponema pedis strain T3552B, T.pedis A17 2004, T.pedis A13 2001, Treponema denticola strain OMZ854, Treponema denticola strain KCOM3500, Treponema putidum strain OMZ758ATCC700334, Treponema putidum strain ATCC700334,Treponema putidum strain OMZ846,Treponema putidum strain OMZ847,Treponema putidum strain OMZ835, Treponema denticola strain OMZ898,Treponema denticola strain CD-1,Treponema denticola strain Marseille-5-CS,Treponema denticola strain OMZ852,Treponema denticola strain OMZ910,Treponema denticola strain OMZ905,Treponema denticola strain OMZ853,Treponema denticolaATCC35405,Treponema denticola strain OKA3,Treponema denticola strain OMZ823,Treponema denticola strain OT2B,Treponema denticola ASLM,Treponema denticolaF0402, Treponema denticola strain ATCC700771, Treponema denticola AL-2,Treponema denticola strain OMZ850 and Treponema denticola strain ATCC700768

Figure 8c:

Circular plot of Treponemataceae Phy2 A13 genome compared with additional available Treponema genomes via CGView comparison tool (CCT). CDS in forward and reverse directions for Treponemataceae Phy2 A13 genome are indicated in the outer rings, and BLAST-based comparisons to additional reference treponema genomes are presented as inner rings in order of highest to lowest similarity to Treponemataceae Phy1 A13 genome. The innermost comparison ring begins with Treponematace_phy2_A17, Treponema pedis strain KS1, Treponema pedis strain T3552B, T.pedis A17, T.pedis A13, Treponema denticola strain OMZ854, Treponema denticola strain KCOM3500, Treponema putidum strain OMZ758ATCC700334, Treponema putidum strain ATCC700334,Treponema putidum strain OMZ846,Treponema putidum strain OMZ847,Treponema putidum strain OMZ835, Treponema denticola strain OMZ898,Treponema denticola strain CD-1,Treponema denticola strain Marseille-5-CS,Treponema denticola strain OMZ852,Treponema denticola strain OMZ910,Treponema denticola strain OMZ905,Treponema denticola strain OMZ853,Treponema denticolaATCC35405,Treponema denticola strain OKA3,Treponema denticola strain OMZ823,Treponema denticola strain OT2B,Treponema denticola ASLMsupercont1.2,Treponema denticolaF0402supercont1.12,Treponema denticola strain ATCC700771,Treponema denticola AL-2,Treponema denticola strain OMZ850 and Treponema denticola strain ATCC700768.

.
